# A systematic review of prevalence of pain in nursing home residents with dementia

**DOI:** 10.1186/s12877-023-04340-z

**Published:** 2023-10-10

**Authors:** Anne-S. Helvik, Sverre Bergh, Kjerstin Tevik

**Affiliations:** 1 Department of Public Health and Nursing, Faculty of Medicine and Health Sciences, Norwegian University of Science and Technology (NTNU), Trondheim, Norway; 2 Norwegian National Centre for Ageing and Health, Vestfold Hospital Trust, Tønsberg, Norway; 3 Research Centre for Age-Related Functional Decline and Disease, Innlandet Hospital Trust, Ottestad, Norway

**Keywords:** Behavioral assessment, Care homes, Daily pain, End of life, Long-term care facilities, Nursing home, Presence of pain, Persistent pain, Residential aged care settings

## Abstract

**Background:**

The prevalence of dementia in nursing home (NH) residents is high, and pain is a troublesome symptom for them. Several studies since 2010 have focused on pain in NH residents with dementia, but there is a lack of systematic reviews on the prevalence of pain in NH residents with dementia.

**Aim:**

To systematically review observational studies published from 2010 to 2023 on how pain is assessed and prevalence of pain found in NH residents with dementia.

**Methods:**

A systematic search was conducted in the MEDLINE, PubMed, PsycINFO, Embase, CINAHL, AgeLine, and Cochrane databases for studies published from January 2010 to August 2023. Studies were included if they were observational studies with a quantitative design where self-report, staff assessment, and/or chart review were used to define the prevalence of pain in samples or subsamples of NH residents with dementia.

**Results:**

Of 184 studies considered, 25 were included. The studies assessed pain as daily, present, clinically relevant, chronic, intermittent, persistent pain and/or if pain affected quality of life. The prevalence of pain was high in most studies of NH residents with dementia independent of whether pain was reported as presence of pain or clinically relevant pain, but the prevalence varied from 8.6% to 79.6%. This prevalence was quite stable across the NH stay, but higher towards the end of life (up to 80.4%). Study designs and methodologies differed considerably. About half relied on an observational assessment inventory.

**Conclusion:**

The number of studies focusing on pain in NH residents with dementia was restricted and methodologies differed considerably. Relatively few studies used an observational assessment inventory. In view of the fact that residents with dementia may have difficulties communicating pain, clinicians should pay attention to pain in these residents, systematically and reliably uncover pain by use of observational inventories, and subsequently treat pain to secure high quality care.

**Supplementary Information:**

The online version contains supplementary material available at 10.1186/s12877-023-04340-z.

## Introduction

Up to 85% of nursing home (NH) residents have dementia, and the severity of dementia in NH residents has increased over the years [1–4]. In Europe and the USA, the majority of people with dementia are in a NH at the time of death [5, 6]. In the present study, we use the term NH, although other studies may use terms such as residential aged care settings, care homes, or long-term care facilities to describe equivalent situations.

Pain is a common symptom in NH residents with dementia. Internationally, studies have found the prevalence of pain to be up to 80% in NH residents with dementia, but this prevalence varies considerably [7–14]. The lowest prevalence of pain in NH residents with dementia was 12% [14]. Some studies have documented the prevalence of pain at admission [7, 15], others independent of the length of stay [16, 17], and some were conducted during the last period of life [18]. However, to the best of our knowledge, there is a lack of systematic reviews that sum up and compare international studies published after 2009 on the prevalence of pain in NH residents with dementia [12].

Pain is not only an unpleasant experience for NH residents with dementia, but may have negative consequences, such as reduced physical functioning [19–21], depression [11], anxiety [11], agitation [22], and aggression [11] as well as limiting social interactions [19], and poorer quality of life [15, 23, 24].

Pain in NH residents with dementia is often linked to medical co-morbidities, particularly musculoskeletal conditions [19] and long-term neuropathic conditions such as diabetes [25, 26]. Furthermore, the experience of pain may be affected by neuropathological changes in the brain due to dementia that has its origins in white matter lesions and grey matter atrophy [19, 27, 28]. The literature provides some evidence that dementia subtype affects pain experience [29]. It is reported that residents with severe dementia more often have pain than those with less severe dementia [7], but the findings are inconsistent [30].

The design and methodologies used to assess prevalence of pain in NH residents with dementia may differ. The assessment of pain in observational studies may include self-reported pain inventories [11], use of a proxy (staff) assessment inventory (behavioral-observational assessment inventories) [31], or collection of information regarding pain documented in medical or clinical records/charts [13, 32]. Studies may also assess the proportion of diagnoses related to pain [11]. Although self-reported pain may be the gold standard for measuring the prevalence of pain, dementia complicates this assessment, because dementia impairs memory and reduces the ability of the resident to verbally communicate pain [28]. NH residents with moderate to severe dementia may not reliably answer questions regarding pain [11]. In these stages, dementia-specific pain assessment inventories undertaken by health care staff that rely on observation of pain and detection of pain-related behavior can be helpful [28, 33]. There are a considerable number (> 15) of behavioral-observational pain assessment inventories for residents with cognitive impairment/dementia [34] that are available for use in NHs. These inventories assess typical pain behavior, such as facial expressions (e.g., frowning, grimacing, rapid blinking), verbalization/vocalization (e.g., crying, gasping, moaning, sighing, calling out), and defense postures (e.g., freezing, tensing, guarding, pushing, crouching), which may be prominent signs of pain in people with dementia [33, 35–37]. Residents with dementia may respond to pain treatment not only with reduced pain but also with less severe neuropsychiatric symptoms [38].

Pain is an indicator used for measuring quality of care in some NHs [39–41]. Furthermore, pain treatment in residents with dementia is demanding [28, 33], but in the recent years there has been a change in focus of pain treatment [28, 31, 42], and the numbers of studies exploring treatment to reduce pain both with and without analgesics [31, 43] have increased. The prevalence and intensity of pain reported in interventions studies of NH residents may differ from the general NH resident population since the included samples may be quite selected. Even so, such studies may contribute to improved pain treatment and lower the prevalence of pain also in NH residents with dementia over the years. The characteristics of NH residents with dementia may shift with changing demographics [1–4] and thus also the prevalence of pain in these residents. Assessment of pain and the validity of the prevalence of pain found may additionally be impacted by working conditions and staffing [44] which are shifting with time.

This review may contribute to a better understanding of characteristics of NH residents with dementia and pain. Furthermore, it may detect the use of several definitions and assessment methods used to define and assess pain at different stages of the NH-stay.

A systematic review of the prevalence of pain in NH residents with dementia as reported in observational studies may provide information relevant for policy makers, health care service management, and professionals in clinical practice [45]. Such a systematic review should also pay attention to how pain was defined and assessed. This information can facilitate for pain assessment in clinical practice as well as the non-pharmacological and pharmacological treatment of pain in NH residents with dementia [44]. Thus, the aim of this study is to systematically review how pain was defined (as daily, present, clinically relevant, chronic, intermittent, persistent and/or affecting quality of life) and the prevalence of pain found in observational studies published from 2010 to 2023 where self-report, staff assessment, and/or chart review were used to define the prevalence of pain in NH residents with dementia.

## Materials and methods

The PRISMA 2020 statement was used as a guideline for writing this review [46]. A PRISMA checklist is provided in S1 Table. We have not registered or published a protocol for this systematic review.

### Search strategy and study selection

Two librarians set up, discussed, and conducted a systematic, computerized search in the MEDLINE, PubMed, PsycINFO, Embase, CINAHL, AgeLine, and Cochrane databases for articles published from January 2010 to August 2023. The last search was performed on 24^rd^ August, 2023. The following search were performed in PubMed: (((((pain[MeSH Terms]) or (pain measurement[MeSH Terms]) or (pain*[Title/Abstract])) AND ((prevalence[MeSH Terms]) or (prevalence[Title/Abstract]))) AND ((dementia[MeSH Terms]) or (“alzheimer disease”[MeSH Terms]) or (dement*[Title/Abstract] OR alzheimer*[Title/Abstract])) AND ((nursing home*[MeSH Terms]) or (nursing home*[Title/Abstract]) OR (care home*[Title/Abstract]) or (residential facilities[MeSH Terms]) or (facilities, residential[MeSH Terms]) or (homes for the aged[MeSH Terms]) or residential age care[Title/Abstract]) or long term care facility*[Title/Abstract])) AND ((English[Filter]) AND (2010:2023[pdat]))). S2 Table provide an overview of the searches performed in the databases.

Articles were exported to and managed using EndNote Version 20. In addition, the reference lists of the included studies were screened to find studies that were not detected in the systematic searches.

Studies were included in the review if the following criteria were met:

- Observational studies with a quantitative design (longitudinal or cross-sectional),
- Study participants had dementia and were living in a NH setting,
- Pain was reported by use of self-reported, staff assessment, and/or chart review,
- Published in a scientific referee-based journal and written in English.

Studies were excluded from the review if they were:

- Theoretical, qualitative, editorial articles, or comments on studies,
- Studies with samples selected to interventions,
- Overview articles, non-systematic review studies,
- Studies without sub-group analyses of NH residents with dementia.

### Identification of relevant studies

After a thorough search each study’s title and abstract were screened by the first and last author (ASH & KT) to determine potential eligibility. The full-text versions were obtained if it was unclear whether the study met the inclusion criteria. The same two authors read all full-text articles and uncertainty regarding study eligibility was resolved through discussion between two of the authors (ASH & KT).

### Data extraction

The first author (ASH) extracted first the information from the eligible studies regarding the year of publication, year of data collection, study country, study population/sample, study design, number of participants, age and gender of participants, inclusion criteria, how pain was assessed, and the time point and time frame for assessment. This information was checked and controlled by the last author. A list and description of the procedures used in the original articles are included as tables in this review.

### Quality assessment

Studies were assessed for quality according to nine predefined criteria (see Table 1) [47, 48] independently by two of the authors (ASH & KT). Disagreement was resolved by discussion between these two authors. A score of 1 was given for + (criteria present), and a score of 0 was given for both – (minus, criteria absent) and ? (? = unclear if criteria was present). The sum score of the quality assessment of each study could vary between 0 and 9.

**Table 1:** Criteria for assessing quality of included studies

**Criteria**		**Score**
1	Clearly described study aims/objectives.	+/−/?
2	Description of inclusion and exclusion criteria/ study participant rates.	+/−/?
3	Description of study population (age and gender).	+/−/?
4	Contained information about study setting.	+/−/?
5	Number of participants with dementia > 200.	+/−/?
6	Information about non-responders versus responders.	+/−/?
7	Funding sources or conflicts of interest that may affect the authors’ interpretation of the results described or ruled out.	+/−/?
8	Ethical approval or consent of participants granted.	+/−/?
9	Includes a discussion of risk of bias in individual studies.	+/−/?

+ (criteria present) = score 1; – (minus, criteria absent) = score 0; ? (unclear if criteria was present) = score 0

An overall methodological quality was calculated. Studies that scored ≥ 8 points of the maximum 9 obtainable points were considered to be of strong quality, studies with a score of 7 points were considered of good quality, fair quality of those with 5 or 6 points, and poor quality when the score was ≤ 4 points [49].

### Ethics

Ethical approval was not required because the study used secondary data.

## Results

### Literature search and selection

**Fig. 1:**
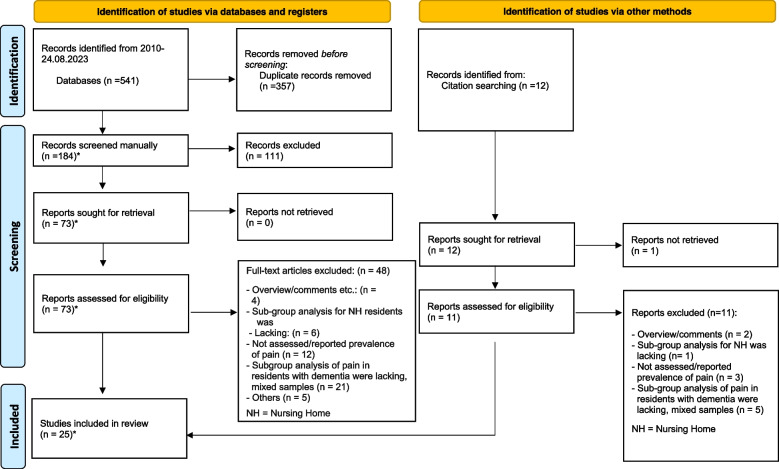
Flow diagram depicting the records that were identified, screened, assessed for eligibility, and the full-text articles reviewed and included in this review [46] *Read and assessed by first and last author (ASH & KT)

The database search identified 541 records. After duplicates were removed 357 records), 184 records remained. Each title and abstract of the 184 records were screened by two authors (ASH & KT), and the full texts of 73 records were considered for possible inclusion. Of these, 25 articles were included. We found 12 additional records in the reference lists of included articles that were not detected through the systematic searches. One of those could not be retrieved, and the rest did not fit the inclusion criteria. Final number of included articles were 25. Figure 1 presents the PRISMA 2020 flow diagram [46], which provides an overview of the search strategy and detailed information about articles that were identified, screened, and assessed for eligibility, and articles included in the review.

### Settings and samples

Table 2 presents the characteristics of the included studies (*N* = 25). The sample size of individual studies ranged from 42 to 3,611,744 NH residents with dementia. The mean age of the participants ranged from 81 to 89 years. All studies included both men and women.

**Table 2:** Articles reporting prevalence of pain in nursing home residents with dementia

**Author, Year,** **Country**	**Aim of the study**	**Participants with dementia: numbers, age, gender**	**Design**	**Inclusion**	**Assessment of pain**	**Prevalence of pain**
Andrews et al. 2019 [32] Australia	To investigate prevalence of pain in NHs residents with dementia	*N* = 114 WD Mean (SD) age = 86.2 (8.1) years Female 71.9%	Cross-sectional	Admitted more than 3 months ago Dementia is diagnosed or suspected after assessment with PAS	Documentation: Pain present when documented in last 3 months Pain was recorded in nursing or medical documentation	Independent of length of stay 86% had at least one documented pain episode the last 3 months
Atee et al. 2021 [16] Australia	To investigate the prevalence of pain in NH residents with NPS by dementia diagnosis	*N* = 479 WD Mean (SD) age = 81.9 (8.3) years Female 55.5%	Cross-sectional	Independent of when admitted Dementia was recorded Has received special service for NPS last year	PainChek Pain present when PainChek ≥ 7 Staff assessment	Independent of length of stay Presence of pain was 65.6% Presence of pain in subsamples: AD (*n* = 196): 64.3% DUN (*n* = 159): 66% VaD (*n* = 61): 62.3% MD (*n* = 28): 78.6% DLB (*n* = 14): 78.6% FTD (*n* = 11): 54.6% Others WD (*n* = 10): 60%
Barry et al. 2015 [50] Northern Ireland/United Kingdom	To investigate the prevalence of pain in NH residents with dementia	*N* = 42 WD Mean (SD) age = 82.1 (7.4) years Female 57.1%	Cross-sectional	Admitted more than four weeks ago Diagnosed with dementia	VDS Pain present right ‘now’ and ‘on an average day’ when VDS > 0 Self-reported, staff assessment and next of kin assessment	Independent of length of stay Presence of pain right now Self-reported: 23.8% Staff assessed: 42.9% Next of kin assessed: 57.1% Pain on an average day: Self-reported: 38% Staff assessed: 69% Next of kin assessed: 75% Missing self-reported pain in about 30% of residents for both assessments
Bunker et al. 2022 [51] USA	To investigate prevalence of pain and pain impacting QoL the last days of life in NH residents with dementia, also seen by financial model for care	*N* = 115,757 Age and gender distribution is not stated Total with any pain *N* = 20,585 N with pain by model of care TM *N* = 13,256 Mean age = 85.9 years Female 67.5% MA *N* = 4909 Mean age = 86 years Female 66.6% ACO *N* = 2420 Mean age = 86.4 years Female 68%	Cross-sectional	Last 30 days of life Dementia diagnoses on MDS Cognitive impairment with CFS score 2 or higher	MDS 3.0 Pain reported present in a 5-day period by resident Any pain present Pain impacting QoL = self-reported pain either on pain that made it hard to sleep or limited the day-to-day activities in a 5-day period	Last 30 days of life Presence of any pain (*n* = 20,585) 17.8% Presence of pain impacting QoL in residents with pain (*n* = 4528) 22% Presence of pain impacting QoL in subsamples residents with pain: 21.6% in TM 22.1% in MA 23.6% in ACO
Dube et al. 2020 [52] USA	To investigate pain in NH residents without cancer at admission	Moderate dementia *N* = 1,973,550 Age ≥ 75 years = 75.8% Female 59.5% Severe dementia *N* = 1,638,194 Age ≥ 75 years = 81.8% Female 62.8% Total sample *N* = 8,613,080 Age ≥ 85 years = 34% Female = 62.5%	Cross-sectional	Newly admitted (assessed ≤ 14 days) Dementia if cognitive impairment was moderate or severe defined by BIMS or CPS	MDS 3.0 Pain reported present in a 5-day period Reported by resident or by staff for residents unable to self-report	Admission Moderate dementia Presence of self-reported pain (*n* = 1,777,495): 49.8% Presence of staff-assessed pain (*n* = 196,055): 41.5% Severe dementia Presence of self-reported pain (*n* = 1,238,621): 32.9% Presence of staff-assessed pain (*n* = 399,573): 37.2%
Estabrooks et al. 2015 [39] Canada	To investigate the symptom burden, including pain, in older NH residents with and without dementia in their last year of life analyzed by work environment characteristics in the facilities	*N* = 2635 WD Age and gender distribution were not stated Total sample *N* = 3647 Mean (SD) age 88 years Female 65.8%	Longitudinal Follow-up: 4 assessments last year of life	Having 4 quarterly RAI-MDS 2.0 reports before death Dementia recorded in the assessment history	RAI-MDS 2.0 Daily pain in a 7-day period that was moderate or worse Reported by resident or by staff for residents unable to self-report	Last year of life In high ranked NH work environments: 4, 3, 2 quarters before death: 14% had daily pain 1 quarter before death: 18% had daily pain In low ranked NH work environments: 4, 3, 2 quarters before death: 17–18% had daily pain 1 quarter before death: 21% had daily pain
Forrester et al. 2021 [53] USA	To investigate pain in NH residents who are unable to self-report pain due to dementia	*N* = 26,816 WD Age ≥ 75 years = 82.3% Female 72.4%	Longitudinal Follow-up 3 & 6 months Pain reported once, baseline	Newly admitted (assessed ≤ 14 days) Residents who are unable to self-report pain due to dementia/cogni-tive impairment	MDS 3.0 Presence of pain detected in a 5-day period Staff assessment	Admission 35.9% had presence of pain In those with stable, severe, or worsening cognitive impairment at 6 months: 38.7% had pain at admission while in those with stable mild/moderate cognitive impairment or some improvement after 6 months 35.3% had pain at admission
Griffioen et al. 2019 [31] Norway	To investigate the prevalence of pain and opioid use in NH residents with dementia and agitation	*N* = 327 WD Mean (range) age 85.7 (65–104) years Female 73.7%	Cross-sectional	Admitted more than four weeks ago Diagnosed with dementia using DSM-IV Having moderate to severe dementia defined by FAST Agitation defined by CMAI	MOBID-2 Pain Scale Clinically relevant pain when MOBID-2 ≥ 3 Staff assessment	Independent of length of stay 62.1% had clinically relevant pain In NH-residents with dementia and pain: 61.6% were prescribed analgesics and 24.6% of those with pain used strong opioids 10.5% without pain also used opioids (not including codeine combinations and tramadol)
Haasum et al. 2011 [54] Sweden	To investigate pain and use of analgesics in older adults with and without dementia living in NH/institution and own home	*N* = 186 WD in NH/ institution Mean (SD) age 89.1 (6.1) years Female 87.1%	Cross-sectional	Independent of length of stay Diagnosed with dementia using DSM-IV	Self-reported pain Experience of any pain last 4 weeks Presence of pain-related diagnoses	Independent of length of stay 8.6% had presence of pain 42.5% had missing or don’t know 48.4% had a pain-related diagnosis
Helvik et al. 2021 [15] Norway	To investigate pain in people with dementia admitted to NH	*N* = 953 WD Mean (SD) age 84 (7.5) years Female 64.2%	Cross-sectional	Newly admitted (≤ 4 weeks) Diagnosed with dementia using ICD-10 criteria	MOBID-2 Pain Scale Clinically relevant pain when MOBID-2 ≥ 3 Staff assessment	Admission 35.5% had clinically relevant pain
Helvik et al. 2022 [55] Norway	To investigate use of analgesics stratified by clinically relevant pain at admission and 12 month and 24 months in NH residents with dementia	N = 996 WD Mean (SD) age 84.5 (7.6) years Female 63.9%	Longitudinal Follow-up: Annual for 2 years	Newly admitted (≤ 4 weeks) Diagnosed with dementia using ICD-10 criteria	MOBID-2 Pain Scale Clinically relevant pain when MOBID-2 ≥ 3 Staff assessment	Admission 35.6% had clinically relevant pain Thereafter 1 year: 37.7% 2 years: 41.5%
Hendriks et al. 2015 [7] The Netherlands	To investigate the course of symptoms, including pain, from admission to death in NH residents with dementia	*N* = 327 WD Mean (SD) age 84 (7.0) years Female 70%	Longitudinal Follow-up: Semiannual assessments for maximum 3.5 years	Newly admitted (8 weeks) Diagnosed with dementia by NH physician	Documentation Pain present when documented frequency ≥ 5 of 30 previous days the second month after admission & average of documented frequency ≥ 5 days of 30 days the three last months before semi-annual assessments Persistent presence of pain when presence of pain at two consecutive assessments Incidence of pain: No presence of pain at one assessment and presence at next Resolution of pain: Presence of pain at one assessment and not at next	Admission 52% had presence of pain Thereafter ½ year: 61% 1 year: 68% 1½ year: 58% 2 years: 56% 2½ years: 47% Last ordinary assessment before death: 67% Persistent presence of pain varied between 36–41% across consecutive assessments Incidence of pain varied between 6–24% across consecutive assessments Resolution of pain varied between 10–13% across consecutive assessments
Hendriks et al. 2014 [18] The Netherlands	To investigate prevalence of symptoms, including pain, in the last week of life in NH residents with dementia	*N* = 330 WD Mean (SD) age at death 85.2 (7.4) years Female 67%	Cross-sectional	Independent of length of stay prior to final week Diagnosed with dementia	Documentation Pain present when documented frequency > 1 day the last week of life	Last week of life 52% had presence of pain
Holmerová et al. 2018 [56] Czech Republic	To investigate pain and use of analgesics in NH residents	Mild dementia *N* = 85 Mean (SD) age 85.6 (7.8) years Female 77.6% Moderate dementia *N* = 171 Mean (SD) age 85.3 (7.4) years Female 83.6% Total sample *N* = 404 Mean (SD) age 84.8 (7.5) years Female 78%	Cross-sectional	Independent of when admitted Mild and moderate to severe dementia defined by MMSE	EQ-5D-3L-pain/ PAINAD Mild dementia Pain present if one item in EQ-5D-3L > 1 Self-reported Moderate to severe dementia Pain present when PAINAD > 0 Staff assessment	Independent of length of stay Mild dementia 54.1% had presence of pain Moderate to severe dementia 39.2% had presence of pain
Hunnicutt et al. 2017 [57] USA	To investigate intermittent pain and persistence of pain in long stay NH residents	WD: Number, age, and gender distribution is not stated Total sample *N* = 1,387,405 Age and gender distribution not stated	Longitudinal Follow-up: 90 days after inclusion	Long term stay (> 100 days) Dementia if cognitive impairment was moderate or severe defined by BIMS or CPS	MDS 3.0 Pain present when reported ≥ 1 of 5-previous days Reported by resident (VDS/NPS > 0) or by staff for residents unable to self-report Intermittent pain present if pain on either of the two assessments Persistent pain present if pain at both assessments	Long term stay Moderate dementia 19.8% had intermittent presence of pain 18.4% had persistent presence of pain Severe dementia 16.9% had intermittent presence of pain 10.5% had persistent presence of pain
van Kooten et al. 2017 [58] The Netherlands	To investigate prevalence of pain in NH residents with dementia	*N* = 199 WD Mean (SD) age 84.9 (6.5) years Female 77.4%	Cross-sectional	Independent of when admitted Living in a Dementia Special Care unit diagnosed with dementia Information regarding dementia subtypes	MOBID-2 in all & NRS/VDS/FPS-R/PAINAD in some In all residents: Clinically relevant pain when MOBID-2 ≥ 3 Staff assessment Residents able to self-report pain (*n* = 122): Clinically relevant pain when NRS ≥ 4, VDS moderate or higher and/or FPS-R third face Residents not able to self-report pain (*n* = 67): Clinically relevant pain when PAINAD ≥ 2 Staff assessment	Independent of length of stay 43.0% had clinically relevant staff assessed pain (MOBID-2) Prevalence of clinically relevant pain in subsamples (MOBID-2): AD (*n* = 106): 41.7% MD (*n* = 31): 38.7% VaD (*n* = 20): 60.0% Others WD (*n* = 42): 41.5% Pain was more prevalent in those with severe dementia (MOBID-2) Prevalence of pain using a self-report inventory or PAINAD was lower (22.1% and 26.9%, respectively) than assessed with MOBID-2
Koppitz et al. 2015 [13] Switzerland	To investigate symptoms noted in records of NH residents with dementia in their dying phase	*N* = 65 WD Mean (SD) age 83.7 (8.5) years Female 75.4%	Longitudinal Follow-up: over 90 days, 4 phases; 90–61, 60–31, 30–8, 7–0 days to death	Living in a Dementia Special Care unit	Documentation Pain present when documented ≥ 1 of 7 days, ≥ 1 of 30 days, ≥ 1 of 90 days	Last 90 days of living 71% had documentation of presence of pain Over the four survey periods (*n* = 33–41): The prevalence of pain increased from 64.2% to 80.4% in the subsamples with detailed period information
Malara et al. 2016 [11] Italy	To investigate prevalence of pain in NH residents with verified dementia	*N* = 181 WD Mean (SD) age male 80.7 (9.3) years Mean (SD) age female 85.6 (7.3) years Female 66.3%	Cross-sectional	Independent of when admitted Diagnosed with dementia using DSM-IV	NRS/PAINAD/ICD-9 in all residents Pain present when NRS > 0 Self-report of pain & PAINAD ≥ 2 Staff assessment Chronic pain documented according to ICD-9-CM	Independent of length of stay 79.6% had presence of self-reported pain among those with reliable answers of NRS (42.5% of total sample) 51.8% had presence of staff assessed pain (PAINAD) 46.4% had chronic pain (ICD-9-CM)
Miu and Chan 2014 [59] Hong Kong	To investigate prevalence of pain in NH residents in residents with dementia	*N* = 309 WD Mean (SD) age 85.0 (7.5) years Female 59.5% Residents with pain: *n* = 190 Mean (SD) age 84.6 (7.6) years Female 65.3% Residents without pain: *n* = 119 Mean (SD) age 85.6 (7.4) years Female 67.2%	Cross-sectional	Admitted more than four weeks ago A diagnosis of dementia from medical record	PAINAD Pain present when PAINAD ≥ 2 Staff assessment	Independent of length of stay 61.5% had presence of pain
Morrison et al. 2020 [60] USA	To investigate prevalence of pain among newly admitted NH residents	Moderate dementia *N* = 268,167 Age ≥ 75 years = 74.2% Female 58.4% Severe dementia *N* = 544,400 Age ≥ 75 years = 75.5% Female 61.6% Total sample *N* = 1,036,806 Age ≥ 75 years = 71.5% Female 60.3%	Cross-sectional	Newly admitted (≤ 14 days) Dementia if cognitive impairment was moderate or severe defined by BIMS or CPS	MDS 3.0 Presence of signs of pain behavior ≥ 1 of the previous 5 days Staff assessment	Admission Moderate dementia 42.4% had presence of pain Severe dementia 38.4% had presence of pain
Raikumar et al. 2017 [17] United Kingdom	To investigate the prevalence of pain in NH residents with dementia at two time-points	*N* = 967 WD Those with pain (*n* = 341) Mean (SD) age 85.3 (8.7) years Female 73.6% Those without pain (*n* = 626) Mean (SD) age 84.2 (9.1) years Female 69.0% *N* = 629 at follow-up	Longitudinal Follow-up once after 9 months	Independent of when admitted Dementia if filling the diagnostic criteria for dementia, and CDRS ≥ 1	APS Pain present when APS ≥ 3 Staff assessment	Independent of length of stay First assessment: 35.3% had presence of pain Second assessment 31.3% had presence of pain
Rostad et al. 2017 [61] Norway	To investigate pain and quality of life in NH residents with severe dementia	*N* = 112 WD Median (range) age 84 (68–99) years Female 69%	Cross-sectional	Independent of when admitted Diagnosis of dementia in medical records Lacking capacity to self-report or communicate pain verbally	Doloplus-2 pain scale Clinically relevant pain when Doloplus-2 ≥ 5 Staff assessment	Independent of length of stay 67.9% had clinically relevant pain
Sengupta et al. 2010 [14] USA	To investigate the prevalence of pain by race and dementia in NH residents	WD: Number, age, and gender distribution not stated Total sample *N* = 14,017 Age and gender distribution not stated	Cross-sectional	Independent of when admitted Dementia when documented in medical record	Documentation Pain present when documented frequency ≥ 1 of the previous 7 days	Independent of length of stay White residents: 18% had presence of pain Non-white residents: 12% had presence of pain
Sjölund et al. 2021 [62] Sweden	To investigate the prevalence of pain in NH residents using different pain assessments and by cognitive impairment	*N* = 95 WD Age and gender distribution were not stated Total sample *N* = 213 Mean (SD) age 85.4 (6.9) years Female 68.5%	Cross-sectional	Admitted more than four weeks ago Dementia is stated by MMSE	Doloplus-2/NRS Pain present when Doloplus-2 ≥ 5 Staff assessment & NRS > 0 Staff assessment	Independent of length of stay 72.6% had presence of pain using Doloplus-2 83.2% had presence of pain using NRS
Tan et al. 2016 [63] Australia	To investigate pain and use of analgesics in NH residents with and without dementia	*N* = 169 WD Mean (SD) age 87.4 (6.1) years Female 78.1%	Cross-sectional	Independent of when admitted Dementia: no information about criteria for labeling dementia	FPS-R/PAINAD Pain present when FPS-R > 0, Self-reported & PAINAD > 0, Staff assessment	Independent of length of stay 66.3% had self-reported presence of pain (FPS-R) 26.0% had staff-assessed presence of pain (PAINAD) High number of missing responses in FPS-R

*ACO* Accountable care organizations, *AD* Alzheimer’s disease, *APS* Abbey Pain scale, *BIMS* The Brief Interview of Mental status, *CDRS* Clinical Dementia Rating Scale, *CFS* Cognitive function score, *CMAI* Cohen-Mansfield Agitation Inventory, *CPS* Cognitive Performance Scale, *DLB* Dementia with Levy bodies, *DSM-IV* Diagnostic and Statistical Manual of Mental Disorders, 4^th^ edition, *DUN* Dementia Unspecified or unknown, *EQ-5D-3L-pain* Euro Quality of life groups questionnaire, one item regarding pain, *FAST* Functional Assessment Staging Test, *FPS-R* Face Pain Scale Revised, *FTD* Frontotemporal Dementia, *GDS* Global Deterioration Scale, *ICD-9-CM* International Classification of Diseases, Nineth Revision, Clinical Modification, *MA* Medicare Advantage, *MD* Mixed Dementia, *MDS* Minimum Data Set, *MOBID-2* Mobilization-Observation-Behaviour-Intensity-Dementia-2, *MMSE* Mini-Mental State Examination, *N* Number, *NH* Nursing Home, *NPS* Neuropsychiatric Symptoms, *NRS* Numeric Rating Scale, *PAINAD* Pain Assessment in Advanced Dementia, *PainChek* Artificial intelligence-based pain assessment inventory, *PAS* Personal Care Assistants, *QoL* Quality of Life, *RAI* Resident Assessment Instrument, *SD* Standard Deviation, *TM* Traditional Medicare, *VaD* Vascular Dementia, *VDS* Verbal Description Scale, *WD* With Dementia

In total, 16 of 25 studies included only NH residents with dementia. Nine studies recruited both NH residents with and without dementia. In total, 14 studies were conducted in Europe, seven in North America, three in Australia, and one in Asia.

#### Design

Seven studies had a longitudinal design [7, 17, 39, 53, 55, 57, 58], and 18 studies had a cross-sectional design.

#### Quality assessment

Table 3 provides a description of the quality assessment of the included studies. Seven studies received ≥ 8 points, indicating strong quality, 10 studies received 7 points (good quality), six studies received 5 or 6 points (fair quality), and two studies received 3 or 4 points (poor quality). In total, 14 of 25 (56%) studies had discussed risk bias appropriately.

**Table 3:** Quality assessment of included articles

	**First author (reference)**	1 Aim	2 Inclusion/ exclusion	3 Population (age/ gender)	4 Study setting	5 Residents with dementia n > 200	6 Responders / non-responders comparison	7 Funding resources/ conflict of interest	8 Ethical approval/ consent	9 Risk of bias discussed	Score ± /? Max score 9
1	Andrews [32]	+	+	+	+	-	-	+	+	?	6
2	Atee [16]	+	+	+	+	+	-	+	+	+	8
3	Barry [50]	+	+	+	+	-	-	+	+	+	7
4	Bunker [51]	+	+	?	+	+	-	+	+	+	7
5	Dube [52]	+	+	+	+	+	-	+	+	?	7
6	Estabrooks [39]	+	?	-	+	+	-	+	+	-	5
7	Forrester [53]	+	+	+	+	+	-	+	+	-	7
8	Griffioen [31]	+	+	+	+	+	-	+	+	+	8
9	Haasum [54]	+	+	+	+	-	+	+	+	+	8
10	Helvik [15]	+	+	+	+	+	-	+	+	+	8
11	Helvik [55]	+	+	+	+	+	+	+	+	+	9
12	Hendriks a [7]	+	+	+	+	+	-	+	+	+	8
13	Hendriks b [18]	+	+	+	+	+	-	+	+	+	8
14	Holmerová [56]	+	+	+	+	+	-	+	+	-	7
15	Hunnicutt [57]	+	+	-	+	+	-	+	+	+	7
16	van Kooten [58]	+	+	+	+	-	-	+	+	+	7
17	Koppitz [13]	+	+	+	+	-	-	+	+	-	6
18	Malara [11]	+	?	+	+	-	-	-	+	-	4
19	Miu and Chan [59]	+	+	+	+	+	-	-	+	?	6
20	Morrison [60]	+	?	+	+	+	-	+	+	+	7
21	Raikumar [17]	+	+	+	+	-	-	+	+	-	6
22	Rostad [61]	+	+	+	+	-	-	+	+	?	6
23	Sengupta [14]	+	?	-	+	+	-	-	-	?	3
24	Sjölund [62]	+	+	+	+	-	-	+	+	+	7
25	Tan [63]	+	+	+	+	-	-	+	+	+	7

+ = score 1; – (minus) = score 0; ? (unclear) = score 0

### Assessment of pain

Staff assessment was used in 14 of the studies to explore prevalence of pain in all residents [11, 15–17, 31, 50, 53, 55, 58–63], while 4 studies used both self-report and staff assessment, depending on the severity of dementia and ability to communicate [39, 52, 56, 57] (Table 4). An additional 5 studies reported the prevalence of pain after studying documentation of pain in the resident’s medical journal and nursing documents [7, 13, 14, 18, 32]. Four of the studies that used staff assessments of all residents also used self-report screening inventories [11, 50, 58, 63], while one of these studies [50] also asked one next of kin to report the residents’ pain. All studies assessed prevalence of pain both in residents with and without pain treatment, thus the studies reflect the number of residents experiencing pain. Residents with pain treatment not experiencing pain at the assessment time point were categorized as not with pain.

**Table 4:** Inventories used to study pain

	**References**
**Self-report**	
FPS-R	[58, 63]
NRS	[11, 58]
VDS	[50, 58]
MDS 3.0	[51]
No inventory reported	[54]
**Staff assessment**	
APS	[17]
Doloplus-2	[61, 62]
NRS	[62]
MOBID-2	[15, 31, 55, 58]
MDS 3.0	[53, 60]
PAINAD	[11, 58, 59, 63]
PainChek	[32]
VDS	[50]
**Combination of self-report and staff assessmen**t	
RAI-MDS 2.0	[39]
MDS 3.0	[52, 57]
EQ-5D-3L-pain combined with PAINAD	[56]
**Next of kin assessment**	
VDS	[50]
**Documentation of pain**	
Medical or nursing documentation reporting pain or pain treatment	[13, 32]
All available records including information about pain	[14, 18]
Documentation audit, no details specified	[7]
**Diagnostic procedure documented**	
ICD-9-CM for chronic pain	[11]

*APS* Abbey Pain scale, *EQ-5D-3L-pain* Euro Quality of life groups questionnaire, one item regarding pain, *FPS-R* Face Pain Scale Revised, *ICD-9-CM* International Classification of Diseases, Nineth Revision, Clinical Modification, *MDS* Minimum Data Set, *MOBID-2* Mobilization-Observation-Behaviour-Intensity-Dementia-2, *NRS* Numeric Rating Scale, *PAINAD* Pain Assessment in Advanced Dementia, *PainChek* Artificial intelligence-based pain assessment inventory, *RAI-MDS* Resident Assessment Instrument Minimum Data Set, *VDS* Verbal Description Scale

The studies that included staff assessment of pain most often used PAINAD and MOBID-2, with 5 studies using PAINAD [11, 56, 58, 59, 63] and 4 studies using MOBID-2 [15, 31, 55, 58]. In total, 4 different self-report screening inventories were used (Table 4). Two studies used both Face Pain Scale Revised (FPS-R) [63] and Verbal Description Scale (VDS) [50, 58]. One study did not report use of a known self-report screening inventory [54].

Pain was reported as the presence of pain, clinically relevant pain, daily pain, chronic pain, intermittent pain, persistent pain, and/or pain impacting quality of life (Table 5). The definitions used to determine the presence of pain and clinically relevant pain varied considerably. Presence of pain had 17 different definitions, while clinically relevant pain had six definitions. Also, the definitions of presence of pain and clinically relevant pain overlapped in some studies [11, 58, 59].

**Table 5:** Pain categories and procedures used to assess pain

**Presence of pain**	**Assessment method**	**Author**
APS ≥ 3	Staff assessment	[17]
PainChek ≥ 7	Staff assessment	[32]
PAINAD > 0	Staff assessment	[56, 63]
PAINAD ≥ 2	Staff assessment	[11, 59]
Doloplus-2 ≥ 2	Staff assessment	[62]
FPS-R ≥ 0	Self-reported	[63]
VDS > no pain	Staff assessment, self-reported	[50]
NRS > 0	Self-reported Staff assessment	[11, 62]
EQ-5D-3L pain item > 1	Self-reported	[56]
MDS 3.0 ≥ 1 of 5 previous days	Staff assessment Combination of self-report and staff assessment Self-reported	[51–53, 57, 60]
≥ 1 of 4 previous weeks	Self-reported	[54]
≥ 1 of 7 previous days	Documented evidence	[13, 14]
> 1 of 7 days the last week of life	Documented evidence	[18]
≥ 5 of 30 previous days the second month after admission	Documented evidence	[7]
≥ 1 of 30 previous days	Documented evidence	[13]
≥ 5 of 30 days a month the three last months	Documented evidence	[7]
≥ 1 of 90 previous days	Documented evidence	[13, 32]
**Clinically relevant pain**		
MOBID-2 ≥ 3	Staff assessment	[15, 31, 55, 58]
Doloplus-2 ≥ 5	Staff assessment	[61]
PAINAD ≥ 2	Staff assessment	[58]
NRS ≥ 4	Self-reported	[58]
VDS ≥ moderate	Self-reported	[58]
FPS-R ≥ third face	Self-reported	[58]
**Daily Pain**		
RAI-MDS 2.0 Daily pain over 7 days	Combination of self-report and staff assessment	[39]
**Chronic pain**		
ICD-9-CM diagnoses	Diagnostic procedure	[11]
Diagnoses and analgesics related to pain	Self-reported	[54]
**Intermittent pain**		
Presence of pain at one of two time points (MDS 3.0 ≥ 1 of 5 previous days, assessment 3 months apart)	Combination of self-report and staff assessment	[57]
**Incidence**		
No presence of pain at first time period and presence at next (average of ≥ 5 of 30 days a month the three last months, assessment 6 months apart)	Documented evidence	[7]
**Resolution**		
Presence of pain at first time period and not at next (average of ≥ 5 of 30 days a month the three last months, assessment 6 months apart)	Documented evidence	[7]
**Persistent pain**		
Presence at two consecutive time periods (average of ≥ 5 of 30 days a month the three last months, assessment 6 months apart)	Documented evidence	[7]
Persistent pain present if pain at both assessments (MDS 3.0 ≥ 1 of 5 previous days, assessment 3 months apart	Combination of self-report and staff assessment	[57]
**Pain impacting Quality of life**		
Presence of pain impacting activities and/or sleep (MDS 3.0 ≥ 1 of 5 previous days on one or two specific items)	Self-reported	[51]

*APS* Abbey Pain scale, *EQ-5D-3L-pain* Euro Quality of life groups questionnaire, one item regarding pain, *FPS-R* Face Pain Scale Revised, *ICD-9-CM* International Classification of Diseases, Nineth Revision, Clinical Modification, *MDS* Minimum Data Set, *MOBID-2* Mobilization-Observation-Behaviour-Intensity-Dementia-2, *NH* Nursing Home, *NRS* Numeric Rating Scale, *PAINAD* Pain Assessment in Advanced Dementia, *PainChek* Artificial intelligence-based pain assessment inventory, *RAI-MDS* Resident Assessment Instrument Minimum Data Set, *VDS* Verbal Description Scale

There were also differences in the time points at which the prevalence of pain was assessed during the NH stay, including shortly after admission (14 days to 8 weeks) [7, 15, 17, 52, 53, 55, 60], after 100 days or more [57], semiannually [7], annually [55], in the last year of life [39], in last 90 days of life [13], last 30 days of life [51] and during the last week of life [13, 18]. However, the prevalence of pain was most often explored in residents with dementia independent of their length of stay, if they met the basic inclusion criteria [11, 14, 16, 17, 31, 32, 50, 54, 56, 58, 59, 61–63]. Five of the studies reported prevalence of pain by stage of dementia, mild [56], moderate [52, 57, 60], severe [52, 57, 60] and/or moderate/severe [31, 56] dementia.

### Prevalence of pain

Most studies that explored the prevalence of pain in residents with dementia independent of length of stay found a high prevalence of pain, but this varied from 8.6% self-reported in residents with dementia in Sweden [54] to 79.6% self-reported among Italian NH residents with a reliable self-report answer [11]. The prevalence of pain was then either assessed as clinically relevant [31, 58, 61], or present in the near term or at the time of assessment [11, 14, 16, 17, 50, 54, 56, 59, 62, 63] (Table 1). One study reported a very high prevalence of pain of 86% [32] as at least one pain episode present the last 3 months, independent of length of stay.

**Fig. 2:**
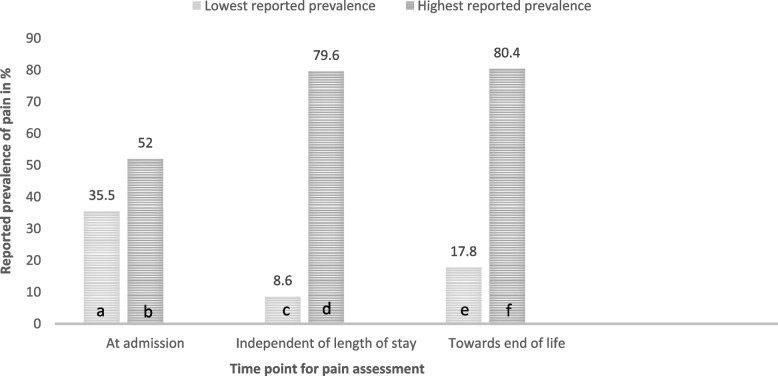
Lowest and highest prevalence of pain reported in the original studies, by the time point of assessing pain during the nursing home stay. Results reported independent of definition of pain, procedure used to assess pain, and severity and type of dementia in the nursing home residents with dementia participating. a = Ref [15], b = Ref [7], c = Ref [54], d = Ref [11], e = Ref [51], f = Ref [13]

The prevalence of pain among NH residents with dementia shortly after admission [7, 15, 52, 53, 55, 60] was between 35.5% [15] and 52% [7], either reported as clinically relevant or as the presence of pain (Table 1, Fig. 2).

The prevalence of pain during the last period of life when assessed as daily pain was between 14–21% in the last year of life [39]. Towards the end of life, reports of both daily pain and the presence of pain were higher, with the prevalence of daily pain as high as 21% and the presence of pain as high as 80.4% [7, 13, 18, 51].

The prevalence of pain was quite stable over time both when residents with dementia were included shortly after admission [7, 55] and included with varying length of stay at baseline [17]. The prevalence of the persistent presence of pain between two consecutive assessments varied from 11–18% in one study [57] to 36–41% in another [7]. In the latter study the incidence and resolution varied between 6–24% and 10–13% across consecutive assessments [7].

The prevalence of pain did not differ among NH residents with Alzheimer’s disease (AD), Vascular Dementia (VAD), and Mixed Dementia (MD) when staff assessments were used to either record pain as present, or as clinically relevant in studies with restricted sample sizes (*n* < 500) [16, 58]. In studies included in the review, the reported prevalence of present pain was somewhat higher in NH residents with mild dementia than in those with more severe dementia in studies that combined self-report and staff assessment inventories, depending on the ability to communicate pain [52, 56, 57]. The prevalence of clinically relevant pain (staff assessment in all residents) was reported in one study and was reported higher in residents with more severe dementia than in residents with mild dementia [58].

The prevalence of pain using self-reported pain inventories was considerably higher than if staff assessed the presence of pain in the same NH residents with dementia [11, 63], but not in all studies [50]. The presence of pain was more prevalent when assessed by the resident’s next of kin with dementia than when self-reported by the resident [50]. The number of NH residents with missing self-report responses [50, 54, 63] or unreliable self-report answers [11] was high.

## Discussion

The prevalence of pain reported in NH residents with dementia varies greatly. Pain was reported as presence of pain, clinically relevant pain, daily pain, chronic pain, persistent/ intermittent pain, or pain affecting quality of life. In the longitudinal studies, the prevalence of a category of pain was quite stable during the NH stay, but higher towards the end of life. Some, but not all, studies found a lower prevalence of pain in NH residents with more severe dementia. There were considerable variations in design, assessments used to report pain, and the procedures used to assess pain as a symptom.

In the six studies exploring pain in NH residents with dementia shortly after admission [7, 15, 52, 53, 55, 60], the reported prevalence was between 36% [15] and 52% [7]. The reported pain prevalence were in some studies assessed as presence of pain one or more times during a single month [7], while other studies either reported pain as present one or more times the last 5 days or as clinically relevant where the intensity of pain also was important [15]. Not surprisingly, the highest prevalence of pain was reported by the study with the longest assessment period and no requirements regarding pain intensity. A study published prior to the time frame of this review reported a prevalence of present pain in the lower range (33.3%) when assessed one or more times during a 5-day period shortly after admission to psychogeriatric wards [64] compared to the results found in the present review [52, 53, 60].

Cross sectional studies of clinically relevant pain in NH residents with dementia independent of their length of stay have reported a somewhat higher prevalence of clinically relevant pain (MOBID-2 ≥ 3) (43–67.9%) [10, 31, 58] than the study reporting clinically relevant pain at admission (MOBID-2 ≥ 3, 36%) [15]. The somewhat higher prevalence of clinically relevant pain independent of length of stay using MOBID-2 is in line with the results from a study investigating clinically relevant pain independent of length of stay using another observational assessment inventory (Doloplus-2 ≥ 5: 67.9%) [61].

Longitudinal studies that reported pain across two or more assessments found the prevalence of present pain in their sample to be quite stable from one assessment to the next [7, 17, 55]. Nevertheless, the prevalence of persistent present pain has been reported restricted. In one study assessing pain 90 days apart in long-term stay residents, the prevalence of persistent presence of pain varied between 11 and 19% [57]. Another study with several consecutive semiannual assessments in residents included at admission reported the prevalence of persistent presence of pain to vary between 36 and 41% [7]. The fluctuation of pain across two or more assessments may partly be explained by the multifactual causes of pain in NH residents [19, 25, 26] and to the dementia itself [19, 27, 28]. In addition, some of the fluctuation may be due to the pain treatment, both pharmacological and non-pharmacological [65], which is an essential part of the care of residents in NH [66].

The present review identified five studies that reported the prevalence of pain in NH residents with dementia during the last period of life [7, 13, 18, 39, 51]. The prevalence of present pain was higher towards the end of life than earlier in the stay [7, 13, 39]. This is in line with an earlier study [67]. The prevalence of pain increased steadily in the last year of life [13], especially during the last phase of life [13, 39]. Patients frequently develop burdensome symptoms during the disease trajectory, which means that adequate symptom and pain control to maintain well-being must be prioritized [19, 68]. However, among residents in USA about 22% of those with pain had a pain impacting quality of life the last 30 days of life [51]. The first step in adequate pain treatment is to uncover undertreated or untreated pain in NH residents with dementia [34, 35].

Even if the gold standard may be self-reported pain, this approach may be problematic as NH residents may have cognitive impairment [28, 62]. Those with dementia may have reduced ability both to respond to questions about pain and verbally communicate their experiences of pain [28]. Thus, a high number of missing responses to self-report questions may be expected, which was also the case for the studies included in the present review [50, 54, 63]. NH residents with moderate to severe dementia may not answers questions regarding pain [11]. One study included in this review that used both a simple self-report and a staff pain assessment in all NH residents independent of severity of dementia found the number of residents with reliable self-reported answers to be quite limited (40%) [11]. The results confirmed that an observational tool is a necessary and suitable way of assessing pain in residents with dementia [11]. The use of dementia-specific pain assessment inventories that rely on health care staff observations and detection of pain-related behavior is highly recommended for residents with moderate to severe dementia [23, 28, 33, 65]. About half of the studies included in this review used an observational assessment inventory to evaluate pain in NH residents with dementia. The most commonly used assessment inventories were PAINAD [11, 56, 58, 59, 63] and MOBID-2 [15, 31, 55]. Both MOBID-2 [69, 70] and PAINAD [71, 72] are considered to be valid and reliable inventories for pain assessment in dementia.

One study using an observational assessment inventory reported a higher prevalence of clinically relevant pain in residents with more severe dementia than in those with less severe dementia [58], which may be expected since the experience of pain may be affected by neuropathological changes in the brain due to dementia that has its origins in white matter lesions and grey matter atrophy [19, 25]. However, in studies combining self-report and staff assessment inventories depending on ability to report or communicate pain, the prevalence of present pain was reported higher in NH residents with mild dementia than in those with more severe dementia [52, 56, 57]. These findings have been explained as assessment flaws in the identification of pain by the staff [52]. Lack of identification of pain has been suggested as the reason why residents in low ranked NH work environments were reported to have a higher prevalence of pain than NH residents living in a highly ranked work environment [39]. It was reasoned that higher ranked work environment detected and appropriately managed pain [39]. Others have reported that the financial model of NH care is linked to the degree of self-reported prevalence of severe pain in residents with present pain [51]. The care model may also explain why a study found that NH residents followed up by their family physician in the NH more often had documented symptoms of pain than NH residents receiving regular care [13]. The care model may also possibly reason why a study found that nonwhite NH residents with dementia to have a lower prevalence of pain than white residents [14].

A mandatory requirement for pain assessment in NH residents, as is mandated in some countries [39–41], is a highly recommended practice for all NH-services. Such assessments need to be followed by strategies to improve the competence and confidence in health personnel in interpreting signs of pain, such as noises, facial expressions, and defense related to body movements in people with dementia [15]. Some studies have recommended an educational program for health professionals in NHs that focuses on how to observe behavioral pain in NH residents with dementia, as well as the use of a systematic pain observational staff assessment and use of a systematic multicomponent pain person-centered treatment approach to assess pain in a reliable way [73–76]. Assessing pain in a reliable way is essential to uncover pain and facilitate non-pharmacological [43] and pharmacological pain treatment [65] and reduce the prevalence of clinically relevant pain in NH residents with dementia, but also to improve their quality of life [15]. Pain should not be overlooked. All NH care practices have to keep in mind that treating pain is an essential component of human care [66] and health professionals have a particular obligation to provide pain treatment when the ability of residents to communicate their pain is reduced or limited [66].

### Strength and limitations

**Fig. 3:**
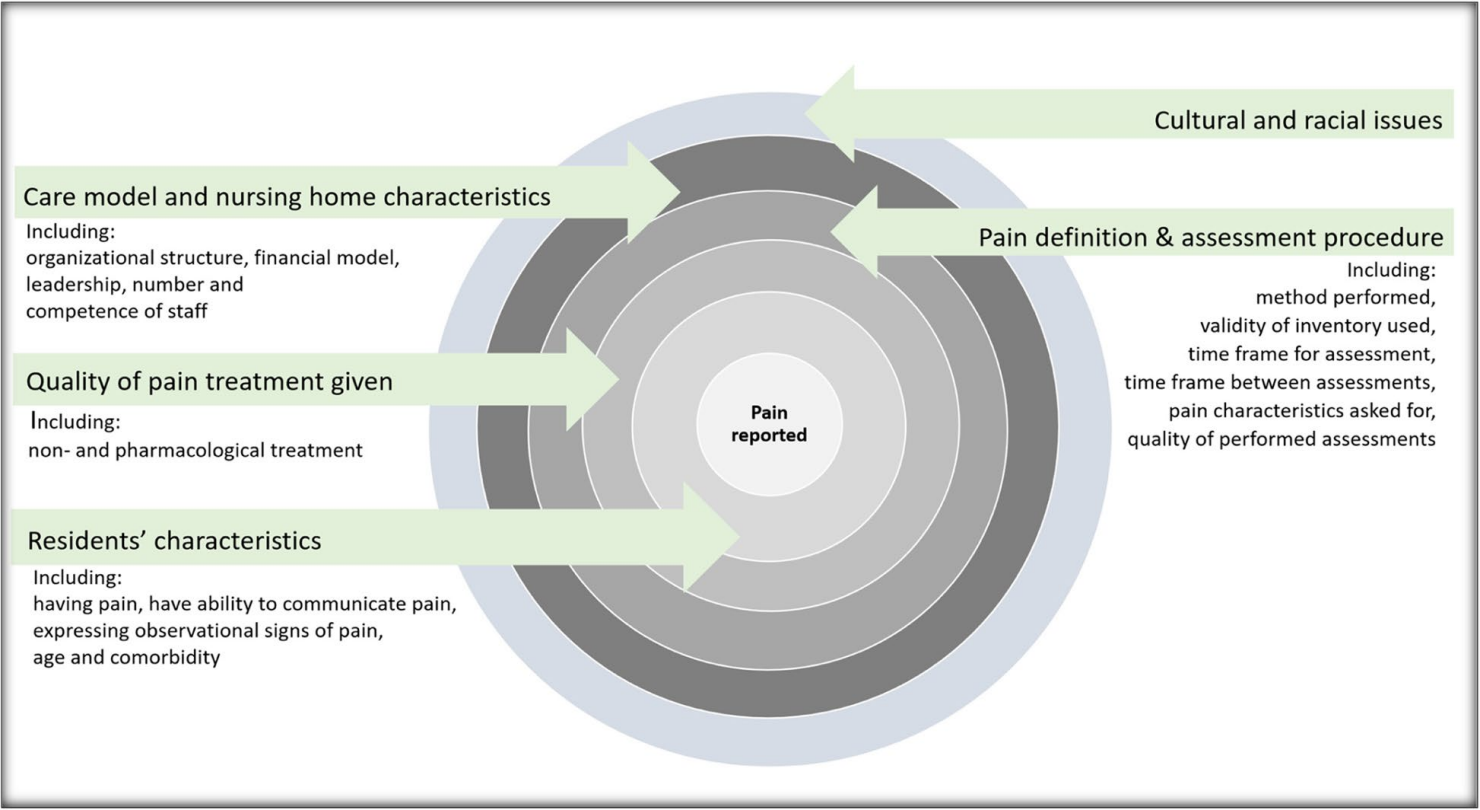
A simplified illustration of factors that may influence pain assessment and reported pain prevalence in nursing home residents with dementia

The major strength of this review is its systematic literature search, the use of several databases, and the careful examination of references in included studies to uncover potential studies not found by the systematic search. Even so, one limitation is that only a few studies use similar definitions and methodologies to assess prevalence of pain. The complexity of the topic makes it difficult combine the data presented in the original studies and perform meta- analyses [77]. The comparison of pain prevalence was organized based on the time frame of the NH resident’s stay and when the assessment was conducted: shortly after admission, independent of time from admission, long-term stay, and last period of life. Furthermore, we compared results reported by category of pain investigated in the published studies and have drawn attention to how pain is assessed, and the inventories used to do so. However, the characteristics of pain defined as daily, present, clinically relevant, chronic, intermittent, persistent, and/or affecting quality of life, the characteristics of pain may overlap considerably. E.g., clinically relevant pain may both be chronic and persistent and also affecting quality of life. It may be hard to reveal the differences in content between some of the terms used. Furthermore, it has been challenging to make comparisons between studies due to sample differences, such as age and gender distribution [34], physical health, and severity of dementia. Differences in health-care systems, or cultural differences among countries, may further complicate a direct comparison between studies when it comes to prevalence of pain [78]. As has been previously discussed in this paper, the specific NH setting characteristics and the services provided may influence pain prevalence reported. Lack of staffing, knowledge, and/or person-centered care may contribute to lack of pain identification and documentation as well as lower validity of the research and pain treatment. Thus, pain information drawn from medical- or nursing records may impact the study quality negatively and contribute to an underestimation of the prevalence. In the present review, only about half of the included studies used an observational assessment inventory. Future studies, exploring prevalence of pain in NH residents with dementia, should, to improve study validity, include an observational assessment inventory to all participants. Cultural and racial differences in expressing pain in NH residents with dementia is another topic for future studies since only one study so far has explored pain by ethnicity [14]. A simplified presentation illustrates factors that may influence pain assessment and reported pain prevalence in NH residents with dementia (Fig. 3).

## Conclusion

There is a high reported prevalence of pain in NH residents with dementia, independent of whether pain was reported as the presence of pain, clinically relevant pain, daily pain, chronic pain, or intermittent pain. The prevalence of pain was quite stable across the NH stay, but higher towards the end of life. There was considerable variation in the methodologies used to report pain in NH residents with dementia. The number of studies using an observational assessment inventory was restricted. Knowing that residents with dementia may have difficulties communicating pain, observational inventories should be used both to uncover and subsequently evaluate the effect of the pain treatment given.

### Supplementary Information

**Additional file 1: S1 Table.** PRISMA 2020 Checklist.

**Additional file 2: S2 Table.** Search in databases.

## Data Availability

All information in the present study is collected from previous published studies, publicly available. All data generated or analysed during this study are included in this published article and its supplementary information files.
