# Supplementary material for: A systematic review of prevalence of pain in nursing home residents with dementia
Source: BMC Geriatr. 2023 Oct 10;23:641. doi: 10.1186/s12877-023-04340-z (PMC10566134; doi:10.1186/s12877-023-04340-z)
Supplement: Supplementary file 2 — Additional file 2: S2 Table. Search in databases. [file 12877_2023_4340_MOESM2_ESM.docx]

S2 Table Search in databases

| Database | Search |
| --- | --- |
| PubMed  110 items | ((("Pain"[MeSH Terms]) or ("Pain Measurement"[MeSH Terms]) or (pain[Title/Abstract])) AND (("Prevalence"[MeSH Terms]) or (prevalence[Title/Abstract]))) AND (("Dementia"[MeSH Terms]) or ("Alzheimer Disease"[MeSH Terms]) or (dement*[Title/Abstract]) or (alzheimer*[Title/Abstract])) AND (("Nursing Homes"[MeSH Terms]) or (nursing home*[Title/Abstract]) or (care home*[Title/Abstract]) or ("Residential Facilities"[MeSH Terms]) or (facilities, residential[MeSH Terms]) or (homes for the aged[MeSH Terms]) or (residential aged care[Title/Abstract]) or ("long term care facilit*"[Title/Abstract] OR "long-term care facilit*"[Title/Abstract])) Filters: English, from 2010 – 2023  The result of search performed in March was 105 items  The updated search in August was 5 items |
| MEDLINE  108 items | 1 (pain adj3 (scale* or tool or rating or measur* or assess* or observat* or identif* or prevalence)).ab,kf,ti. (80345)  2 Pain/ (150710)  3 Pain Measurement/ (94327)  4 pain.ab,kf,ti. (756773)  5 1 or 2 or 3 or 4 (808672)  6 Prevalence/ (340257)  7 prevalence.ab,kf,ti. (773667)  8 6 or 7 (864361)  9 5 and 8 (36694)  10 Dementia/ (60766)  11 Alzheimer Disease/ (116081)  12 (dement* or alzheimer*).ab,kf,ti. (273453)  13 10 or 11 or 12 (291420)  14 Nursing Homes/ (39005)  15 nursing home*.ab,kf,ti. (36033)  16 care home*.ab,kf,ti. (5450)  17 Homes for the Aged/ (14719)  18 Residential Facilities/ (5736)  19 "long term care facilit*".ab,kf,ti. (6978)  20 "long-term care facilit*".ab,kf,ti. (6978)  21 (aged adj3 (residential or home*)).ab,kf,ti. (3910)  22 14 or 15 or 16 or 17 or 18 or 19 or 20 or 21 (71050)  23 9 and 13 and 22 (153)  24 limit 23 to yr="2010 -Current" (112)  25 limit 24 to english language (104)  26 limit 25 to journal article (102)  The result of search performed in March was 102 items  The updated search in August was 6 items |
| PsycInfo  40 items | 1 exp Pain/  2 exp Pain Measurement/  3 pain.ab,id,mh,sh,ti,tm.  4 1 or 2 or 3  5 prevalence.ab,id,mh,sh,ti.  6 4 and 5  7 exp Dementia/  8 exp Alzheimer's Disease  9 (dement* or alzheimer*).ab,id,mh,sh,ti.  10 7 or 8 or 9  11 exp Nursing Homes/  12 residential care institutions/  13 "nursing home*".ab,id,mh,sh,ti.  14 "care home*".ab,id,mh,sh,ti.  15 home* for the aged.ab,id,mh,sh,ti.  16 (long term care facilit* or long-term care facilit*).ab,id,mh,sh,ti.  17 11 or 12 or 13 or 14 or 15 or 16  18 6 and 10 and 17 (  19 limit 18 to yr="2010 -Current"  20 limit 19 to english language  21 limit 20 to "0100 journal"  The result of search performed in March was 39 items  The updated search in August was 1 item |
| Embase  98 items | (pain/ OR pain assessment/ OR pain.ab,kf,ti. AND prevalence/ OR prevalence.ab,kf,ti.)  AND dementia/ OR Alzheimer disease/ OR (dement* or alzheimer*).ab,kf,ti.  AND nursing home/ OR (nursing home* or care home*).ab,kf,ti.  The search in March was 98 items  The updated search in August was 0 items |
| CINAHL  74 items | ((MH "Residential Care+") OR ( aged N1 (facilit* or home*).AB,TI,SU. OR ( long term care facilit* or long-term care facilit*).AB,TI,SU. OR  ( nursing home* or care home* ).AB,TI,SU. OR (MH "Nursing Homes+")) AND ( dement* or alzheimer*).AB,TI,SU. OR (MH "Alzheimer's Disease") OR (MH "Dementia+") AND (Prevalence.AB,TI,SU. OR(MH "Prevalence") AND pain,AB,TI,SU, OR (MH "Pain Measurement") OR (MH "Pain+"))  The search in March was 71 items  The updated search in August was 3 items |
| AgeLine  18 items | ( long term care facilit* or long-term care facilit* )AB,TI,SU OR ( residential N1 (institution* or facilit*)AB,SU,TI OR DE "Board and Care Homes" OR (DE "Nursing Homes" OR DE "For Profit Nursing Homes" OR DE "Nonprofit Nursing Homes" OR DE "Teaching Nursing Homes") OR (DE "Nursing Homes") OR ( nursing home* or care home* ).AB,TI,SU. OR (DE "Nursing Homes")  AND ( dement* or alzheimer*).AB,TI,SU. OR DE "Alzheimers Disease" OR  DE "Dementia" OR DE "Alzheimers Disease" OR DE "Early Onset Dementia" OR DE "Frontotemporal Dementia" OR DE "Lewy Body Dementia" OR DE "Vascular Dementia  AND ((Prevalence.AB,TI,SU. OR (DE "Prevalence")  AND pain,AB,TI,SU, OR Pain Measurement.AB,TI,SU. OR DE "Pain" OR DE "Chronic Pain")) ( nursing home* or care home* ).AB,TI,SU. OR (DE "Nursing Homes")  AND ( dement* or alzheimer*).AB,TI,SU. OR DE "Alzheimers Disease" OR  DE "Dementia" OR DE "Alzheimers Disease" OR DE "Early Onset Dementia" OR DE "Frontotemporal Dementia" OR DE "Lewy Body Dementia" OR DE "Vascular Dementia  AND ((Prevalence.AB,TI,SU. OR (DE "Prevalence")  AND pain,AB,TI,SU, OR Pain Measurement.AB,TI,SU. OR DE "Pain" OR DE "Chronic Pain"))  The search in March was18 items  The updated search in August was 0 items |
| Cochrane  28 items | #1 MeSH descriptor: [Pain] explode all trees  #2 MeSH descriptor: [Pain Measurement] explode all trees  #3 (pain):ti,ab,kw (Word variations have been searched)  #4 #1 or #2 or #3  #5 MeSH descriptor: [Prevalence] explode all trees  #6 (prevalence):ti,ab,kw (Word variations have been searched)  #7 #5 or #6  #8 #4 AND #7  #9 MeSH descriptor: [Alzheimer Disease] explode all trees  #10 MeSH descriptor: [Dementia] explode all trees  #11 (dement* or alzheimer*):ti,ab,kw (Word variations have been searched)  #12 #9 or #10 or #11  #13 MeSH descriptor: [Nursing Homes] explode all trees  #14 (nursing home* or care home*):ti,ab,kw (Word variations have been searched)  #15 MeSH descriptor: [Homes for the Aged] explode all trees  #16 MeSH descriptor: [Residential Facilities] explode all trees  #17 (residential aged care):ti,ab,kw (Word variations have been searched)  #18 (long term care facilit*):ti,ab,kw (Word variations have been searched)  #19 (long-term care facilit*):ti,ab,kw (Word variations have been searched)  #20 #13 or #14 or #15 or #16 or #17 or #18 or #19  #21 #8 AND #12 AND #20  The search in March was 28 items  The updated search in August was 0 items |

The two systematic searches were performed (23.03.2023 and 23.08. 2023), the first search included the period from 2010 to 23th March 2023 and the second search and included the period between 3^rd^ week of March to dd. An identical search string for each database was used in both searches and covered articles published in academic journals using the English language.
